# A Family-Based Lifestyle Intervention Focusing on Fathers and Their Children Using Co-Creation: Study Protocol of the Run Daddy Run Intervention

**DOI:** 10.3390/ijerph18041830

**Published:** 2021-02-13

**Authors:** Julie Latomme, Philip J. Morgan, Marieke De Craemer, Ruben Brondeel, Maïté Verloigne, Greet Cardon

**Affiliations:** 1 Department of Movement and Sports Sciences, Ghent University, 9000 Ghent, Belgium; ruben.brondeel@ugent.be (R.B.); Greet.Cardon@UGent.be (G.C.); 2 PRCPAN (Priority Research Centre for Physical Activity and Nutrition), School of Education, University of Newcastle, Newcastle 2308, Australia; philip.morgan@newcastle.edu.au; 3 Department of Rehabilitation Sciences, Ghent University, 9000 Ghent, Belgium; Marieke.DeCraemer@UGent.be; 4 Department of Public Health and Primary Care, Ghent University, 9000 Ghent, Belgium; maite.verloigne@ugent.be

**Keywords:** study protocol, intervention, physical activity, fathers, children, families, eHealth

## Abstract

Fathers play a unique and important role in shaping their children’s physical activity (PA), independent from the mother. Lifestyle interventions focusing simultaneously on PA of fathers and their children (“co-PA”) are therefore a novel and promising way to improve PA of both. A theory-based lifestyle intervention was co-created with fathers (i.e., the Run Daddy Run intervention), using the behavior change wheel as a theoretical framework. The aim of the present study is to describe the protocol of the Run Daddy Run intervention study, focusing on improving (co-)PA of fathers and children, and the prospected outcomes. The developed intervention consists of six (inter)active father-child sessions and an eHealth component, delivered over a 14-week intervention period. Baseline measurements will be conducted between November 2019–January 2020, post-test measurements in June 2020, and follow-up measurements in November 2020, with (co-)PA as the primary outcome variable. Outcomes will be measured using accelerometry and an online questionnaire. To evaluate the intervention, multilevel analyses will be conducted. This study will increase our understanding on whether a theory-based, co-created lifestyle intervention focusing exclusively on fathers and their children can improve their (co-)PA behavior and has important implications for future research and health policy, where targeting fathers might be a novel and effective approach to improve (co-)PA and associated health behaviors of both fathers and their children.

## 1. Introduction

Low levels of physical activity (PA) can be found in many European primary-school aged children. For example, 4.6% to 16.8% of the European primary school-aged children (10–12 years old) does not meet the international recommendation of at least 60 min of moderate-to-vigorous PA per day [1]. This is of concern, as low levels of PA are associated with negative physical and psychological health outcomes, including a higher risk of overweight and obesity [2]. Therefore, promoting PA in children has become an important focus in health promotion and obesity prevention research [3].

It is widely accepted that parents play an important role in establishing healthy lifestyle behaviors in their children, as well as in sports performance [4,5,6]. However, most studies have not distinguished between the differential influence of fathers and mothers on their children’s PA levels [7,8,9]. Yet, recent research indicates that fathers play a unique and important role in shaping their children’s health behaviors [10,11,12], especially PA. Studies have shown that both fathers’ weight status and PA levels are positively associated with their children’s weight status and PA levels, independently of the weight status and PA levels of the mother [12,13,14,15,16]. Moreover, a recent study including a sample of 934 European father-child dyads has shown that the association between fathers’ weight status and their children’s weight status is mediated by the level of PA of the father, through the level of PA of the child [17]. Indeed, studies have suggested that “active play” between father and child is more central to fathers’ parenting style than it is to mothers [18,19], and that this kind of play is advantageous for the motoric development of the child [16]. Furthermore, this kind of play may not only influence both fathers and children their PA behavior, but also the quality of their relationship and the psychosocial wellbeing of the child [20,21,22]. This suggests that focusing on PA of both the father and the child might be a potentially effective intervention strategy, as previous intervention studies have mostly engaged mothers while fathers have not been targeted [23,24]. A possible strategy could be to focus on “co-PA”, where the father and the child participate in PA together. Whereas mothers tend to play a larger role in the logistical planning of PA, fathers are more likely to engage in modelling PA [25,26]. Fathers spend more time with their child in unstructured, competitive, wrestling-style play (i.e., also referred to as ‘rough-and-tumble’ play), which is a form of play that is more vigorous and with greater physicality than that of mothers [25,26]. The modelling of vigorous and moderate-to-vigorous PA by fathers has been shown to be associated with higher levels of PA in their children [26,27].

According to our knowledge, only two programs have been developed specifically targeting fathers and children, which were both Australian. Healthy Dads Healthy Kids aimed to help overweight fathers lose weight and establish positive health behaviors for their children [10,28], whereas the DADEE (Dads and Daughters Exercised and Empowered) program aimed to promote PA between father and daughters [29,30] The results of these two Australian interventions showed that engaging fathers and children in co-PA increased both fathers’ and children’s (total) PA, positively influenced their weight status, improved the father-child relationship and the social-emotional well-being of the child. Further research is however necessary to better understand the effects of co-PA, and to confirm the abovementioned results in a European context. Using an experimental approach is thereby highly recommended [31,32]. Furthermore, it is not only important to investigate the effect of the intervention on (co-)PA of both fathers and children, but also the factors that determine (co-)PA, for example the parenting practices of the father, psychosocial determinants of (co-)PA such as attitude, self-efficacy and knowledge regarding (co-)PA and the family health climate regarding PA in the family. Literature has already shown that these factors play an important role, but were not yet sufficiently targeted in previous interventions [16].

Last, it is highly recommended that an intervention is grounded in and informed by a theoretical framework, in order to result in larger effect sizes [33,34]. Additionally, most lifestyle interventions do not seem to meet fathers’ needs and preferences [35], which results in low participation rates [36,37]. Therefore, the perspective of the fathers should be taken into account when developing an effective intervention [38]. Recently, there has been increased attention for using a co-creation approach in health promotion intervention studies [39,40,41]. The process of co-creation, which may be defined as a strong and active collaboration between end-users (i.e., fathers) and researchers [42], might be a promising approach to enhance effectiveness [43,44].

Taken together, this paper presents the study protocol for a co-created theory-based family-based lifestyle intervention (i.e., the Run Daddy Run intervention) whose main goal is to improve (co-)PA of fathers and their children, as well as other outcomes that are related to (co-)PA (i.e., its psychosocial determinants, sedentary behaviour (SB), parental practices, body Mass index (BMI), quality of the father-child relationship and the family health climate regarding PA). This study will increase our understanding on whether a theory-based, co-created lifestyle intervention focusing exclusively on fathers and their children can improve their (co-)PA and associated health behaviors. This has important implications for future research and health policy, where targeting fathers might be a novel and effective approach to improve health and health behaviors in children. The items addressed in this protocol paper are based on the 2013 Standard Protocol Items: Recommendations for Interventional Trials (SPIRIT) statement [45]. The completed SPIRIT Checklist has been included as Supplementary File S1. 

## 2. Materials and Methods

### 2.1. Development of the Run Daddy Run Intervention

The Behavior Change Wheel was used as a theoretical framework to systematically develop the intervention [46,47]. The intervention development process, modified and reproduced from ‘A Guide to Using the Behavior Change Wheel’ [47], follows three stages: (1) understanding the behavior, (2) translating findings into an intervention, and (3) refining and pre-testing the intervention, including 10 steps across the three different stages (see Table 1). Additionally, a co-creation approach was included in the intervention development process [41]. An overview of how this co-creation approach was combined with the behavior change wheel is presented in Table 1, where the main goal of each step is also described. The 10 steps were systematically followed where the completion of the tasks in one step created a product that was the guide for the subsequent step. More specifically, two co-creation groups with fathers were carried out. The first co-creation group (i.e., co-creation Group A) consisted of a group of five fathers (for descriptive statistics see Table 2), with whom four co-creation sessions were conducted between September–November 2018. Each of the four sessions lasted about 150 min and were facilitated by two researchers, who received a training by co-creation experts in using a co-creation approach to ensure effective delivery of, and engagement of the fathers during the sessions. Key principles in the development, implementation and evaluation of co-creation projects were followed during the entire development process [39,41]. The content of each of these co-creation sessions and its link with the different steps of the behavior change wheel can be found in Table 1. During these co-creation sessions, the intervention goals were selected and described, which were based on the barriers and motivators experienced by the fathers. In total, 13 intervention goals were described (see Table 3). These intervention goals were then linked by the researcher to the COM-B components and theoretical domains of the Theoretical Domain Framework (TDF) of the Behavior Change Wheel (for an overview see Supplementary File S2)

The intervention “concept” created by co-creation Group A was then presented to and fine-tuned by a second co-creation group (i.e., co-creation Group B), in December 2018. This co-creation group included five fathers with a lower socio-economic status and/or higher BMI (for descriptive statistics see Table 3) who were each separately interviewed to discuss and review, in detail, the end-result of the co-created intervention by Group A. The result of both co-creation groups was the Run Daddy Run intervention, which is a family-based lifestyle intervention, targeting (co-)PA of fathers and their primary-school aged children (6–8 years old) as the primary outcome, as well as its psychsocial determinants, SB, parental practices, BMI, quality of the father-child relationship and the family health climate regarding PA as secondary outcomes. As a last step of the intervention development process, the intervention was pre-tested in a pilot study among a group of 12 fathers and children to evaluate the created intervention for the first time and to make some final adjustments, of which the results will not be reported in the current study protocol.

For the co-creation sessions and interviews, fathers were recruited through convenience sampling, flyers and social media. Requirements to participate were; having at least one primary school-aged child (6–12 years old), speaking Dutch and having a smartphone with internet connection.

After each co-creation session/interview, participants were asked to complete a process evaluation questionnaire, enabling an evaluation of the session/interview given, and ensuring that the key principles of co-creation were met [39]. More specifically, participants were asked to give a score, ranging from 1 (weak) to 5 (excellent) on several dimensions important in co-creation (e.g., climate of trust and openness, equal influence on decision, being able to share opinion, etc.). In general, the mean scores of Group A on the dimensions ranged from 3.75–5 on a total score of 5, and in Group B from 4.25–4.75/5, suggesting that the co-creation sessions and interviews met the key principles of co-creation.

### 2.2. The Run Daddy Run Intervention

The main aim of the Run Daddy Run intervention was to increase (co-)PA by engaging fathers and children together in PA. Additionally, some components were added to target psychosocial determinants of (co-)PA, SB, and other health related components (i.e., PA family context, quality of father-child relationship and parental practices towards PA). The content of the intervention, created during the intervention development process, is described below.

#### 2.2.1. Content of the Run Daddy Run Intervention

**Figure 1:**
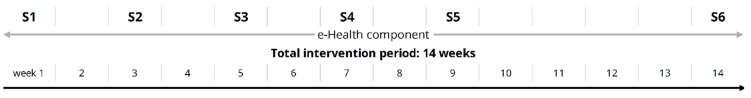
Timeline of the total intervention period.

The intervention consists of a face-to-face component, including six (inter)active sessions for fathers and their children, and an eHealth component. Both components will be delivered over a 14-week intervention period. A timeline of the total intervention period is presented in Figure 1. The sessions for will be delivered on a two-weekly basis, each session lasting 120 min. The last session will be delivered four weeks after the fifth (and thus second last) session, so that this session can serve as a follow-up session. All sessions will be guided by three facilitators (one main facilitator and two supporting facilitators, which are trained experts in movement and sports and/or health promotion sciences). The eHealth component will be used throughout the entire intervention period. Participants will be assigned to three different groups (based on their date- and time preferences), each including about 12 father-child dyads. Each group will receive a session on a different evening in the same week. All sessions will take place at a local school for convenience reasons.

**Figure 2:**
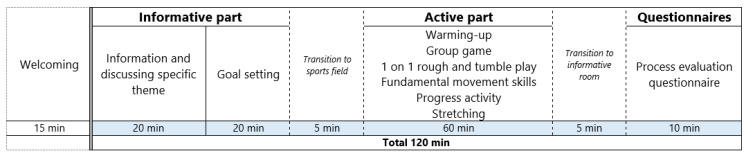
Timeline of an (inter)active father-child session.

Each session consists of a 40-min informative part (i.e., with some education and theory on a particular topic) and a 60-min active part. The timeline of such a session can be found in Figure 2. In the informative part, education and theory will be provided by a facilitator to the fathers on a particular theme that appeared to be important by fathers during the intervention development (see Table 4 for the different themes of the informative part). This will be done using an oral presentation with didactic slides. Children will work separately on that same theme with the other two other facilitators, using a child-friendly method (i.e., coloring task). Thereafter, co-PA goals will be set by the father-child dyads in group. These goals will be SMART: specific, measurable, attainable, realistic and timely (e.g., “on Saturday afternoon we will play soccer in our garden for 20 min”), which will yield a higher change of success in achieving them [48]. After setting these goals, they will be entered on their personal profile on the Run Daddy Run website, which will be described in more detail below.

After the informative part, fathers and children will move to the sports field/hall where the active part of the sessions will take place. The active part of the session (60 min) includes various exercises and activities for fathers and their children. More specifically, each session will include six different exercise components, with each session having two to four fundamental movement skills (FMS) as the main theme throughout the session (see Table 4 for the different themes of the active part). In Supplementary File S3, an overview of the different FMS practiced in each session can be found in more detail. An overview of the last component of the active part (i.e., the progress activity, with the aim to rehearse and perform better on all the FMS learned throughout the sessions) can be found in Supplementary File S4.

**Figure 3:**
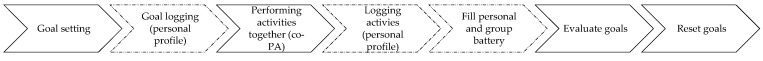
Graphical representation of goal setting and logging process on the profile on the website.

The online part of the intervention consists of a website (www.rundaddyrun.be (accessed on 14 January 2021)) with a profile that can be accessed by all participants with their personal login and password, during the entire 14-week intervention period. A visual representation of this personal platform can be found in Supplementary File S5. As eHealth components of an intervention are more effective when they include one or more behavior-change techniques (BCTs), the online platform will consist of the BCT goal setting, self-monitoring, shaping knowledge and instructions on how to perform the behavior. More specifically, fathers and children will be asked in each session (six times in total) to set a (SMART) co-PA goal, and log it on their personal profile. The aim will be to reach this co-PA goal by the next session, by performing and logging co-physical activities at home (i.e., self-monitoring). For a graphical representation of this logging process, see Figure 3. By logging activities performed together, participants will fill their “personal battery” (i.e., which is full when reaching the personal goal), as well as the “group battery” (i.e., which is full when all individual goals of the group are reached). In each subsequent session, goals will be evaluated and a new, modified goal will be set. The aim of this new goal is to find an optimal balance between achievable and challenging.

A third behavioral change component that is included in the online platform is shaping knowledge, including instructions on how to perform the behavior. More specifically, fathers and children will have access to a large variety of PA and exercise ideas that can be performed together, with concrete instructions. Additionally, also practical information can be found on this personal platform, such as data of the (inter)active sessions, documents and materials used in the sessions. Last, it is recommended in the literature to combine the usage of a website with a reminder system, such as automated emails or text messages to reinforce website use [49] in order to address the high levels of attrition that are negatively affecting many online interventions [50,51]. Therefore, the use of the personal platform on the website will be combined with a chat group in which each group of participants will be included based on their mobile phone number (after explicit permission), together with the facilitators of the (inter)active sessions. The main goal of this chat-message group is to create a positive group atmosphere and group dynamics, and for the facilitators to send reminders for settings goals and logging the performed activities.

#### 2.2.2. Participants

The Run Daddy Run intervention will include Belgian fathers and their primary school-aged children from the first, second and third year of primary school (6–8 years old). The first three years of primary school were targeted in order to optimize group homogeneity regarding cognitive and motor abilities of the children. Furthermore, younger children are more susceptible to behavior shaping compared to older children. The following inclusion criteria will be applied: being the (step) father and/or male primary caregiver of a child of 6–8 years old; being Dutch-speaking; being in good health; and having a mobile phone with internet access.

#### 2.2.3. Sample Size

Using the software GPower 3.0.10 (Universität Kiel, Kiel, Germany) [52], the required sample size was calculated. Based on the obtained effect sizes of previously implemented interventions for fathers and children targeting co-PA [52], a minimally detectable effect size of f = 0.20 was intended for the main PA outcome (i.e., co-PA) in the present study. This effect size was slightly more conservative than the obtained effect sizes for co-PA in the studies of Morgan et al. (i.e., n = 6 reported effect sizes across 3 studies, range f = 0.42–0.73, mean f = 0.58). This sample size calculation was based on 80% power to detect a significant difference in PA outcomes between groups, significance level alpha 0.05, having two groups (intervention and control group) and three measurements (pre-test, post-test and follow-up). The a priori power analysis suggested a total sample size of 42 families (fathers and children). Assuming an attrition rate of 20% [10], a total sample size of 51 fathers will be required.

#### 2.2.4. Recruitment

For the intervention study, fathers and their primary school-aged children (6–8 years old) will be recruited in multiple ways through convenience sampling and snowball sampling. These methods of sampling will be used in order to reach a sufficient number of male participants, as research has indicated that it is difficult to engage fathers for lifestyle interventions [12,53]. More specifically, researchers will recruit fathers through contacting their acquaintances, friends and family. Fathers will also be recruited online, through social media and e-mail. Last, flyers and posters will be distributed in schools, sports clubs, libraries, etc. Additionally, snowball sampling will be used where registered fathers will be asked to contact their friends, family or acquaintances and invite them to participate in the intervention. The recruitment procedures will continue until the required number of participants is reached.

#### 2.2.5. Study Design and Randomization

**Figure 4:**
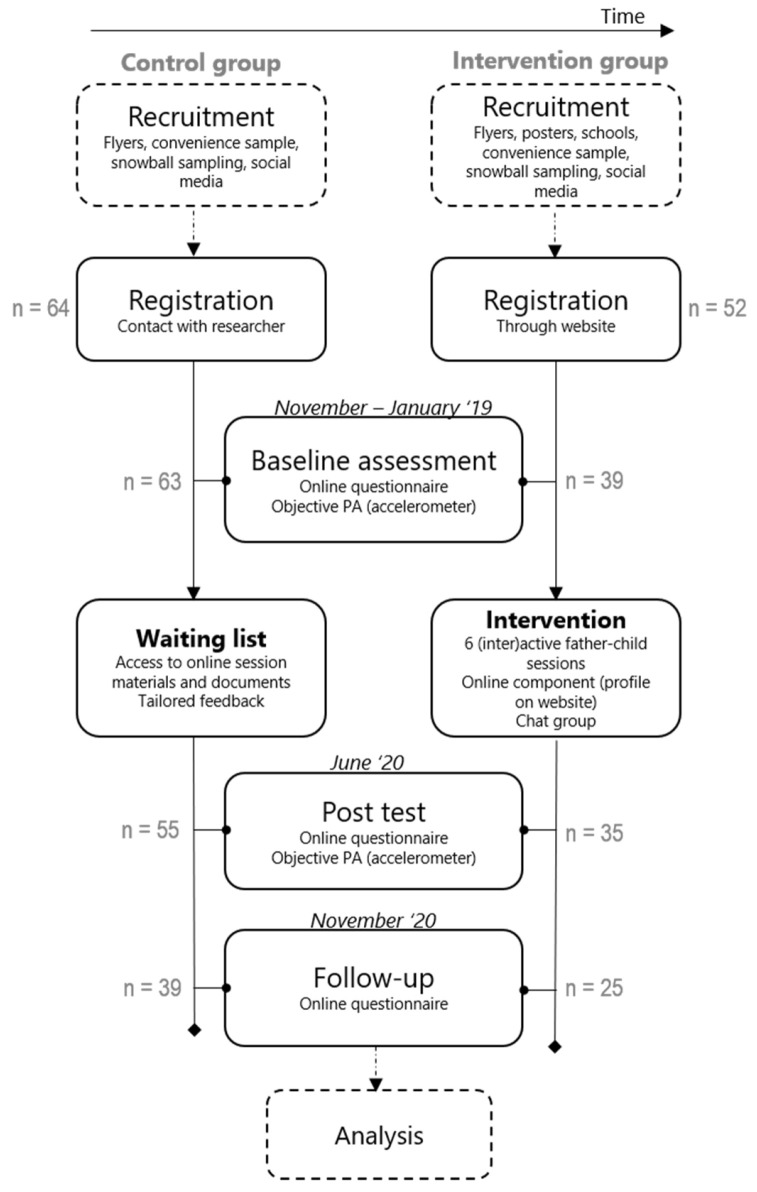
Study design and flow of the Run Daddy Run intervention.

The Run Daddy Run intervention will be evaluated through a quasi-experimental study, with a non-equivalent pre-test post-test control group design in which participants will not be randomly assigned to the control group (CG) or intervention group (IG), but will be recruited subsequently (i.e., first the CG, then the IG—see Figure 4 for a visual representation of the study design and flow). This will be done for convenience reasons and because the study load between the two groups will differ significantly, thus correctly and transparently communicating to the participants what is expected is important for an optimal sample size and to minimize drop-out. The IG will receive the Run Daddy Run intervention, whereas the CG will receive a report of the data acquired with the online questionnaire (i.e., report with information on BMI, PA levels, etc.) after the intervention took place (in June 2020). At that moment, the CG will also get access to all the intervention materials and documents used in the intervention. Measurements will be taken before (i.e., baseline) and after the intervention (i.e., post-test) for both the IG and CG. Additionally, a follow-up measurement will be conducted five months after the intervention.

#### 2.2.6. Procedure

The recruitment phase will take place in November 2019–January 2020. The CG will be asked to participate in a study that investigates health-related behaviors over time, whereas the IG will be asked to participate in a study providing six (inter)active father-child sessions over a total period of 14 weeks. When interested in participating, fathers will be able to register for the study by contacting the researcher (control group) or by registering through an online registration form available on the Run Daddy Run website (intervention group). After registration, the participants will be checked for their eligibility (see Section 2.2.2) and eligible fathers will be asked to complete an online questionnaire and to wear an accelerometer during 7 consecutive days (see Section 2.2.7). The baseline measurements will take place in November-December 2019 for the CG and in January 2019 for the IG. After the completion of the baseline measurements, participants of the IG will receive an email or text message in which they will be informed about the start of the intervention (i.e., date, time and location) and some practical information regarding the first session. In each session, attendance of the father-child dyads will be logged by the facilitators. When a father-child dyad does not attend a certain session, a message (i.e., text message or email) will be sent afterwards with a link to the website where both the information given in the information part and the exercises in the practical part can be found. However, participants who do not attend the first two sessions, will be excluded from the study. Last, post-test measurements will take place after the intervention, in June 2020 and the follow-up measurements will take place in November 2020.

#### 2.2.7. Measurements (Effect Evaluation)

Both subjective and objective measurements will be performed during baseline and post-test, to measure the effect of the intervention. The primary outcome is (co-)PA, all the others are secondary outcomes. During the follow-up measurements, only subjective measurements will be conducted (i.e., online questionnaire but no accelerometry).

##### Co-Physical Activity and Co-Screen Time

Co-PA and co-ST will be measured using a seven-day recall diary, in which fathers will be asked to report all physical activities and screen time activities they performed together with their child in the last seven days. More specifically, fathers have to report the start hour of the activity/activities, duration of the activity/activities, and the activity/activities itself in this diary, for each day of the week. PA diaries are often economical and can provide information on the types of activity not recorded from more objective measurement methods, such as accelerometers [54]. According to Matthews et al. (2002), diary based self-reported instruments can provide, with good participation compliance, accurate and valid assessments of PA-related behaviors [55].

##### Physical Activity

Objective PA data will be collected through accelerometry. Axivity accelerometers (model AX3, 3-axial), which have been shown to be reliable and valid [56], will be worn simultaneously by the father and the child for at least 7 consecutive days, on the non-dominant hand, for 24 h a day. Participants’ light (LPA), moderate (MPA), vigorous (VPA) and total PA will be assessed during this time period, as an additional measure of PA, fathers will be asked to complete the International Physical Activity Questionnaire Short Form (IPAQ-SF), for both himself (i.e., self-report) and his child (i.e., parent-report), questioning LPA, MPA and VPA during the past seven days [57,58]. Research comparing the IPAQ-SF with objective measures (i.e., accelerometers) shows that the criterion validity of this questionnaire is fair to good, with an ICC 0.30 [58]. Overall, the IPAQ-SF has reasonable (test-retest) reliability (ICC = 0.65) a good internal consistency (Cronbach’s alpha = 0.83) [59].

##### Sedentary Behavior

Participants’ total sedentary time per day will be assessed during a seven day period using Axivity accelerometers. Additionally, SB (including ST) will be assessed using the International Sedentary Assessment Tool (ISAT) questionnaire, which will be completed by the father both for himself (i.e., self-report) and his child (i.e., parent-report) [60]. This questionnaire has a good internal consistency (reliability) (Cronbach alpha = 0.80) and good criterion validity (interclass correlation r = 0.63) [61]. In the ISAT, SB is questioned on a typical week- and weekend day (hours/day), as well as specific ST-related behaviors are questioned (i.e., TV/DVD viewing, computer/laptop/PlayStation use and smartphone/tablet use).

##### Body Mass Index

Body mass index (BMI, in kg/m²) of the fathers and children will be calculated based on self-reported (for the father) and parent-reported (for the child) weight and height in the questionnaire. BMI z-scores will be used for children, which is a sex- and age specific measure of their BMI.

##### Determinants of (co-)PA and (co-)ST

Furthermore, 13 (paternal) determinants of health behavior, selected by the research team, will be questioned through a questionnaire. The selection was based on the determinants found in the literature that are possibly related to (co-)PA [62,63,64,65,66], and on the information derived from the co-creation sessions with fathers. The following paternal determinants were questioned to the father: (1) knowledge regarding the PA norm for both adults and (2) children, (3) knowledge regarding PA and (4) self-efficacy towards motivating child for PA, (5) knowledge regarding ST and (6) interrupting SB, (7) attitude towards limiting ST, (8) attitude towards and (9) importance of co-PA, (10) self-efficacy towards increasing co-PA, (11) degree of experienced barriers towards co-PA, (12) social support towards co-PA and (13) habits towards co-PA. For determinants on knowledge, fathers were asked to estimate the norm (in minutes). The deviation from the norm was then calculated, with the value 0 representing a correct estimate of the norm and the higher the score deviating from 0, the greater the deviation from the correct answer. The other determinants were questioned and rated on a numerical response scale (values between 0 and 100, with 0 as the lowest score and 100 as the highest score).

##### PA Family Context, Quality of the Father-Child Relationship and Parental Practices towards PA

The family context regarding PA will be questioned using the Family Health Climate Scale (FHC-PA) [67]. The family health climate can be seen as a determinant of person’s health behavior, and is defined as shared perceptions and cognitions concerning a healthy lifestyle within a family, and represents a family level variable that is intra- and inter-individually correlated to family environmental and individual factors. The FHC-PA is a validated questionnaire containing three subscales: value, cohesion and information on PA in the family. In total, this questionnaire contains 14 items, where participants can score these items on a four-point Likert scale ranging from 1 “definitely false” to 4 “definitely true”. The total score is the sum of all individual items scores, ranging from 14 to 56.

Quality of the relationship with the father will be measured with the nurturant fathering scale (NFS) [68]. This scale is a valid and reliable measure to characterize the relationship between the father and the child [69,70,71]. It consists of 9 items, each rated on a 5-point scale, and possible scores on this measure range from 9 to 45.

Parental skills and practices towards PA will be questioned in 16 questions, derived from four valid and reliable questionnaires each measuring specific parenting practices and skills [72,73,74,75,76]. The validity and reliability of these questionnaires can be found in the corresponding studies mentioned.

#### 2.2.8. Data Analysis

Descriptive statistics will be provided for the sample characteristics, i.e., for the total sample and by study group (i.e., CG and IG). To evaluate the intervention effect (i.e., the difference in pre-post-follow up evolution between control and intervention group), multilevel analyses will be used to take into account the clustering of measurements within participants. Time by group interaction terms will be reported. Age and gender will be considered as confounders in the analyses. For comparison with previous research [10,28], Cohen’s *d* will be reported, which is the difference of two group means divided by the standard deviation from the data. For the process evaluation, the six key elements described in Section 2.2.9 will be quantitatively and qualitatively analyzed. All statistical analyses will be performed using the statistical program SPSS 26.0 for Windows [77].

#### 2.2.9. Data Management

All data will be stored on a password-protected computer and central disk space. Data from the website will additionally be stored on password-encrypted servers. Only persons who are part of the research team will have access to the raw data. Consent forms will be stored separately from participant data, and a unique identifier code will be assigned to each participant. Data will be stored for a maximum of 5 years before being securely destroyed.

#### 2.2.10. Process Evaluation

The use of both qualitative and quantitative data provides the strongest evidence for process evaluation [78]. Therefore, qualitative and quantitative data will be collected in this study. To evaluate the process of the intervention, process evaluation tools were developed based on the recommendations of Saunders, Evans and Joshi [79] who described six important key elements to conduct a process evaluation: (1) fidelity (quality of intervention implementation), (2) dose delivered (extent in which the intervention was delivered, i.e., number of sessions that were attended), (3a) dose received—exposure (active participation level and level of use of the materials and resources), (3b) dose received—satisfaction (satisfaction level of the participants), (4) reach (participation rate), (5) recruitment (followed procedures) and (6) context (barriers and facilitators for implementing the intervention). These key elements will be incorporated in several process evaluation tools (i.e., a process evaluation questionnaire for the fathers, a self-reported observation checklist for the facilitators and log data of the website). The process evaluation component of this study will take place during the intervention delivery (i.e., after each session). An outline of the process evaluation questions, data sources and data collection tools can be found in Supplementary File S6.

### 2.3. Ethics Approval and Consent to Participate

This study was approved by the Committee of Medical Ethics of the Ghent University Hospital (Belgian registration number: B670201941599) and registered as a clinical trial in October 2020 (Clinicaltrials.gov (accessed on 14 January 2021) NCT04590755). Participants of the co-creation sessions and the intervention study received an information letter in which they were briefly informed about the purpose of the study. Informed consents for participants of the co-creation sessions were provided and signed before the start of the sessions/interview. Informed consents for the participants of the intervention study will be provided and signed by the participants several times; once before the online questionnaire is completed, once before wearing the accelerometers and once before participating in the (inter)active sessions (IG only). Each participant will be informed about the design of the study, its purpose, confidentiality of data, and the fact that (s)he has the right to leave the study at any time without stating any reason. Precautions will be taken to ensure participants’ privacy during data analysis. Personal information will be coded and password-protected. All data will be stored on a password-protected computer and central disk space. Data from the website will additionally be stored on password-encrypted servers. Only researchers that are part of the research team will have access to the data.

## 3. Results: Developed Intervention and Prospected Outcomes

The result of the intervention development was a theory-based, co-created lifestyle intervention (i.e., the Run Daddy Run intervention) targeting (co)-PA in fathers and their children as the primary outcome. The aim of this intervention is to improve (co-)PA of fathers and their children, as well as its psychosocial determinants (e.g., attitude, knowledge, self-efficacy towards co-PA), SB, parental practices, weight status (i.e., BMI), the quality of the father-child relationship and the family health climate towards PA. The developed intervention consists of 6 (inter)active father-child sessions and an eHealth component, delivered over a 14-week intervention period, which will start in February 2020 and will end in May 2020. Baseline measurements will be conducted between November 2019–January 2020, post-test measurements in June 2020, and follow-up measurements in November 2020. Important protocol modifications will always be reported on Clinicaltrials.gov (accessed on 14 January 2021). The results of the study will be communicated via various publications.

## 4. Discussion

This paper describes the study protocol including the systematic development of the Run Daddy Run intervention. The main aim of this intervention is to increase (co-)PA in fathers and their primary-school aged children (6–8 years old), in order to prevent childhood overweight and obesity. Additionally, the Run Daddy Run intervention also aims to improve other health-(related) behaviors and outcomes, such as SB, weight status (i.e., BMI) of both fathers and children, and factors that determine (co-)PA, for example the parenting practices of the father, psychosocial determinants of (co-)PA and the family health climate regarding PA in the family. Literature has already shown that these factors play an important role, but were not yet sufficiently target in previous interventions [16].

The behavior change wheel was used as a theoretical framework to develop the intervention, combined with a co-creation approach in which the target group (fathers) was actively involved in the intervention development process to meet their specific needs. The developed Run Daddy Run lifestyle intervention consists of a face-to-face component, including 6 (inter)active sessions over a 14-week intervention period. Additionally, the intervention includes an eHealth component, where fathers and children will have access to a personal platform on a website to log and enter PA goals and activities. To our knowledge, this is one of the first studies that uses the behavior change wheel in combination with a co-creation approach to develop a theory-based intervention specifically for fathers and their children. As previous interventions mainly focused on parents and/or mothers [12,23], this study represents an important contribution to the field of family-based lifestyle interventions. The advantage of using a step-wise theoretical framework is that it ensures that all the necessary theory-based elements for an intervention program are present. Additionally, it has been suggested that using a participatory or co-creational approach may enhance the success of the intervention [80]. The advantage of including co-creation in the development process is that the intervention is not only developed ‘top-down’, but also ‘bottom-up’, including the needs and preferences of the target group. This may help us to develop effective and theory-based interventions for fathers and their children. We hypothesize that the Run Daddy Run intervention will be able to elicit changes in the main outcome variable, i.e., co-PA between fathers and their children. Furthermore, improvements are expected in other health related outcomes such as SB and weight status, and in psychosocial determinants of (co-)PA, parental practices towards PA, quality of the relationship between father and child, and the PA family health climate. These changes in health behaviors and outcomes are expected, as they are targeted in the Run Daddy Run intervention. Outcomes will be measured using accelerometry and questionnaires. To evaluate the effects of the intervention, multilevel analyses will be conducted. Together, this study will contribute to the literature of both the theoretical and practical domain of developing interventions targeting (exclusively) fathers and their children. On a theoretical basis, this paper describes how researchers can co-create an intervention based on a theoretical framework, such as the behavior change wheel. On a practical basis, Run Daddy Run is a theory-based, co-created intervention with different components that can be disseminated on a broader scale if found to be effective.

Limitations include that blinding of the data analyst is not possible since the researcher who will analyze the data is also involved in the data-collection process. To account for this issue, a strict protocol has been developed for processing and analyzing the data. Furthermore, it is possible that despite the advantages of using a convenience sample in this study, this sampling/recruiting method might result in recruiting primarily the most active and motivated fathers and children (i.e., a selection bias). However, this will be taken into account during the recruitment, by mentioning that the goal is to be active together in a playful way, and that it will not only be about vigorous sports activities. By doing so, we aim to make the threshold to participate as low as possible, also for those who are less motivated or active. Furthermore, multiple efforts will be done to recruit a wide range of participants, taking into account the recruitment strategies that emerged during the co-creation sessions with the target group. Another limitation is that our first co-creation group consisted of participants with a higher education and a normal BMI range. Since this may not be a representative sample for the general population, we also included a second co-creation group (i.e., co-creation Group B), which included participants with a lower education and a higher BMI. A last limitation might be the use of self-report measures (e.g., to assess BMI), which can provoke bias. Objectively measuring BMI may overcome this issue, which is therefore recommended for future research.

Strengths of this study are its experimental and longitudinal design (i.e., outcomes are measured at different points in time: pre-test, post-test and follow-up). A second strength is that the intervention includes an eHealth component. eHealth interventions (defined as “the use of information and communication technologies for health” [81]), have emerged as promising and effective for improving PA [50,82,83,84,85], mainly because of its ability to provide efficient, interactive and tailored content to the user [86,87]. However, existing (eHealth) interventions are often not grounded in a theory [86] and the evidence for the effectiveness of these interventions among this target group is still scarce. As the eHealth component in our intervention includes theory-based behavioral change components, it addresses this shortcoming. A last strength is that PA and SB will not only be measured subjectively through questionnaires, but also objectively through accelerometry. This balances another limitation of the fact that self-report measures of PA and SB can lead to response and recall biases [88].

## 5. Conclusions

This study will increase our understanding on whether a theory-based, co-created lifestyle intervention focusing exclusively on fathers and their children can improve (co-)PA and associated health behaviors in fathers and children. This has important implications for future research and health policy, where targeting fathers might be a novel and effective approach to improve health and health behaviors in children

## Figures and Tables

**Table 1:** Different steps of the intervention development process, which is based on the behavior change wheel and co-creation.

Behavior Change Wheel	Researcher	Co-Creation Part (Researcher and Fathers)
Stage 1: understanding the problem describing intervention goals		
Step 1 Identifying the (health) problem, selecting and specifying target behavior	Identify health problem Specify target behavior	/
Step 2 Understanding the problem and identifying what needs to change	List potential barriers and motivators for co-PA based on literature	Co-creation session 1
		Co-mapping (i.e., researcher and fathers mapping together) barriers and motivators for co-PA, which form the basis for the intervention goals Co-mapping recruitment-, user-, and intervention preferences and needs
Step 3 Defining the intervention goals	List potential intervention goals based on barriers and motivators mapped in previous session	Co-creation session 2
		Session 2. Co-selecting and describing intervention goals
Step 4 Selecting COM-B and TDF	Select the COM-B components of the COM-B model, the theoretical domains of the Theoretical Domain Framework and the intervention functions for each of the intervention goals	/
Stage 2: translate findings into intervention		
Step 5 Selecting intervention functions	Select intervention functions	/
Step 6 and 7 Selecting BCTs and practical strategies	Select potential BCTs and practical strategies based on literature and previous experience with interventions	Co-creation session 3
		Co-mapping and selecting BCTs Translating BCTs into practical strategies
Step 8 Integration	Integrate all information of previous steps into one coherent intervention	Co-creation session 4
		Evaluate the integrated intervention proposed by the researcher and refine
Stage 3: refining and pre-testing		
Step 9 Refining	Evaluate the co-created intervention by conducting interviews with another co-creation Group (B)	Co-creation interviews (Group B)
		Refining the intervention created by group A (i.e., by providing feedback, sharing thoughts, …)
Step 10 Pre-testing—pilot study	(out of the scope of this article)	

**Table 2:** Participant characteristics of co-creation Groups A and B.

	Co-Creation Group A	Co-Creation Group B
Number of participants (n)	5	5
Region	West-Flanders, Belgium	East-Flanders, Belgium
Age, in years (range)	36–50	31–47
BMI, kg/m² (range)	21.6–24.6	22.5–32.15
Education		
- 10–12 yrs.	0	1
- 13–14 yrs.	0	1
- 15 yrs. of education (Bachelor degree)	2	1
- ≥16 yrs. of education (Master degree)	3	2
Number of children in the family (range)	2–3	2–3
Age range (yrs.) of all children in the household	7–12	4–14

**Table 3:** Overview of the 13 intervention goals.

Intervention Goals
Making time for co-PA
2. Having social support for co-PA
3. Having insight into the mutual interests for co-PA
4. Having insight into the possibilities/options for co-PA
5. Knowing how to make co-PA a habit
6. Knowing how to positively motivate and communicate with the child during co-PA
7. Limit (co- and individual) screen time
8. Coping with the ‘mental load’ that comes with co-PA (e.g., practical arrangements)
9. Knowing/experiencing the advantages and positive feelings that come with co-PA
10. Having a positive attitude towards co-PA
11. Having insight into the importance of functioning as a positive role model and being engaged in co-PA
12. Learning (motor) skills to their children during co-PA
13. Being motivated for co-PA

**Table 4:** Overview of the different themes derived from the co-creation sessions.

Session Number	Theme	
	Informative Part Theme Based on Identified Barriers for Co-PA According to Fathers	Active Part Theme Based on 2 to 4 FMS
1	General introduction, importance of (co-)PA and role of the father	Jumping, landing, running and coordination
2	Motivation for (co-)PA	Throwing, kicking, catching and rolling
3	Co-PA preferences and common co-PA interests	Rotating, rolling, pulling and pushing
4	Social support	Dribbling and striking
5	Screen time	Carrying, wheedling, crawling and lifting
6	Co-PA as a habit, rehearsal and summary	All FMS

## Data Availability

Data sharing is not applicable to this article as no datasets were generated or analyzed during the current study.

## Supplementary Materials

The following are available online at https://www.mdpi.com/1660-4601/18/4/1830/s1, File S1: completed SPIRIT checklist. File S2: overview of the intervention goals linked with the COM-B model, the TDF framework and BCTs. File S3: overview of the different FMS practiced in the (inter)active sessions. File S4: overview of the parcours of the progress activity including all FMS of the sessions. File S5: personal platform of fathers and children on the Run Daddy Run website. File S6: process evaluation component, with its six key elements.

## Trial Governance

The principal investigators (J.L. and G.C.) are responsible for overseeing the study. The study coordinator (J.M.) is responsible for liaising with study participants, coordinating data collection and data management/storage. R.B., M.D.C., P.J.M. and M.V. will advise on specific aspects of the study including recruitment, practitioner training, data analysis and process evaluation procedures. Any changes to the study protocol will be discussed before the trial registry is updated.

## Trial Registration Number

This study is registered as a clinical trial (clinicaltrials.gov (accessed on 14 January 2021), ID number: NCT04590755).

## Abbreviations

PA	Physical activity
SB	Sedentary behavior
BMI	Body mass index
Co-PA	Co-physical activity
IG	Intervention group
CG	Control group
