# Supplementary material for: A Family-Based Lifestyle Intervention Focusing on Fathers and Their Children Using Co-Creation: Study Protocol of the Run Daddy Run Intervention
Source: Int J Environ Res Public Health. 2021 Feb 13;18(4):1830. doi: 10.3390/ijerph18041830 (PMC7918485; doi:10.3390/ijerph18041830)
Supplement: Supplementary file 1 [file ijerph-18-01830-s001.zip › ijerph-1093862 Supplementary File S2.docx]

**Supplementary File 2 S2.** Overview of the intervention goals linked with the COM-B model, the TDF framework and BCTs.

| **COM-B component** | | **TDF Domain** | **Intervention functions** | **Intervention goal** | **BCT** | |
| --- | --- | --- | --- | --- | --- | --- |
| Opportunity | Physical | *Environmental context and resources* | Education  Persuasion  Incentivisation  Training  Modelling  Enablement | 1. Making time for co-PA | - 1. Goal setting   2. Problem solving   3. Action planning   4. Review behaviour goal(s)   5. Discrepancy between current behaviour and goal   6. Behavioral contract   7. Commitment   8. Instruction on how to perform a behaviour (skills training)   9. Demonstration of the behaviour (modeling)   10. Behavioral practice/rehearsal   11. Credible source   12. Social reward (positive reinforcement)   13. Conserving mental resources   14. Identification of self as role model |  |
|  | Social | *Social influence* | Modelling | 1. Having social support for co-PA | - 1. Problem solving   2. Social support   3. Restructuring the social environment |  |
| Capability | Psychological | *Knowledge* | Education  Training | 1. Having insight into the mutual interests for co-PA | - 1. Social support   2. Instruction on how to perform a behaviour (skills training)   3. Behavioral practice/rehearsal   4. Conserving mental resources   5. Information on emotional consequences   6. Monitoring of emotional consequences   7. Information about others’ approval |  |
|  |  |  | Education  Enablement  Training  Modelling | 1. Having insight into the possibilities/options for co-PA | - 1. Habit formation   2. Monitoring of behaviour by others without feedback   3. Social support   4. Conserving mental resources   5. Instruction on how to perform a behaviour (skills training)   6. Behavioral practice/rehearsal |  |
|  |  | *Behavioral regulation* | Education  Persuasion  . | 1. Knowing how to make co-PA a habit | - 1. Habit formation   2. Generalization of the target behaviour   3. Behavioral practice/rehearsal   4. Credible source   5. Information on health & emotional consequences   6. Graded tasks   7. Social support   8. Conserving mental resources   9. Goal setting   10. Action planning   11. Review behaviour goal(s)   12. Behavioral contract   13. Commitment   14. Self-monitoring of behaviour |  |
|  |  | *Skills* | Education  Persuasion  Training | 1. Knowing how to positively motivate and communicate with the child during co-PA | - 1. Action planning   2. Social support   3. Instruction on how to perform a behaviour   4. Information about health consequences   5. Information about emotional consequences   6. Demonstration of the behaviour   7. Behavioral practice/rehearsal   8. Habit formation   9. Generalization of a target behaviour   10. Credible source   11. Identification of self as role model   12. Framing/reframing   13. Vicarious consequences |  |
|  |  |  | Education  Restriction | 1. Limit (co- and individual) screen time | - 1. Problem solving   2. Self-monitoring of behaviour   3. Information about health consequences   4. Credible source   5. Identification of self as role model |  |
|  |  | *Memory, attention and decision processes* | Education  Training | 1. Coping with the ‘mental load’ that comes with co-PA (e.g. practical arrangements) | - 1. Reduce negative emotions   2. Stress management   3. Conserving mental resources   4. Problem solving |  |
| Motivation | Reflective | *Beliefs about consequences* | Education  Persuasion  Incentivisation  Modelling | 1. Knowing/experiencing the advantages and positive feelings that come with co-PA | - 1. Problem solving   2. Conserving mental resources   3. Information on health and emotional consequences   4. Salience of consequences   5. Credible source   6. Monitoring of emotional consequences   7. Verbal persuasion about capability   8. Exposure   9. Social reward/incentive |  |
|  |  |  | Persuasion  Modelling | 1. Having a positive attitude towards co-PA | - 1. Problem solving   2. Social support   3. Information on health and emotional consequences   4. Salience of consequences   5. Credible source   6. Monitoring of emotional consequences   7. Verbal persuasion about capability   8. Exposure   9. Social reward/incentive   10. Conserving mental resources |  |
|  |  | *Professional role/identity* | Education  Persuasion  Incentivisation  Training  Modelling | 1. Having insight into the importance of functioning as a positive role model and being engaged in co-PA | - 1. Instruction on how to perform a behavior   2. Information about health and social consequences   3. Monitoring of emotional consequences   4. Social comparison   5. Behavioral practice/rehearsal   6. Habit formation   7. Credible source   8. Social incentive   9. Identification of self as role model   10. Verbal persuasion about capability |  |
|  |  |  | Education  Training  Modelling | 1. Learning (motor) skills to their children during co-PA | - 1. Instruction on how to perform the behaviour   2. Demonstration of the behaviour   3. Social comparison   4. Behavioral practice/rehearsal   5. Exposure   6. Identification of self as role model |  |
|  |  | *Optimism* | Education  Persuasion | 1. Being motivated for co-PA | - 1. Goal setting   2. Action planning   3. Commitment   4. Self-monitoring of behaviour   5. Social support   6. Information on health consequences   7. Monitoring of emotional consequences   8. Modelling   9. Habit formation   10. Social incentive |  |
