# Supplementary material for: A Family-Based Lifestyle Intervention Focusing on Fathers and Their Children Using Co-Creation: Study Protocol of the Run Daddy Run Intervention
Source: Int J Environ Res Public Health. 2021 Feb 13;18(4):1830. doi: 10.3390/ijerph18041830 (PMC7918485; doi:10.3390/ijerph18041830)
Supplement: Supplementary file 1 [file ijerph-18-01830-s001.zip › ijerph-1093862 Supplementary File S3.docx]

**Supplementary File S3**. Overview of the different FMS practiced in the (inter)active sessions

| **Session number** | **Fundamental Movement Skills** | **Stations** | **Materials** |
| --- | --- | --- | --- |
| 1 | Running, jumping & landing | S1: Jumping & landing  S2: Jumping & landing  S3: Running & coordination  S4: Sprinting | Silicone lines  Skipping rope  Rope ladder  Timer |
| 2 | Catching, throwing & kicking | S1: Throwing and scoring  S2: Kicking and scoring  S3: Throwing, catching and rolling  S4: Throwing | Ball  Ball  Ball  Frisbee |
| 3 | Rotating, pulling & pushing | S1: Rotating, kicking and catching  S2: Rolling  S3: Rotating  S4: Pulling & pushing | Zip bags  Somersaults  Cartwheels  Push-ups & squads |
| 4 | Striking & dribbling | S1: Dribbling with hands  S2: Striking  S3: Striking  S4: Dribbling with foot | Basket ball  Ball(oon)  Badminton racket  Ball |
| 5 | Wheedling, crawling, lifting & carrying | S1: Carrying  S2: Wheedling & crawling  S3: Lifting & cairrying  S4: Wheedling & crawling | Pingpongball & racket  Ropes  /  / |
| 6 | All above mentioned FMS (rehearsal): group game | | |
