# Supplementary material for: A Family-Based Lifestyle Intervention Focusing on Fathers and Their Children Using Co-Creation: Study Protocol of the Run Daddy Run Intervention
Source: Int J Environ Res Public Health. 2021 Feb 13;18(4):1830. doi: 10.3390/ijerph18041830 (PMC7918485; doi:10.3390/ijerph18041830)
Supplement: Supplementary file 1 [file ijerph-18-01830-s001.zip › ijerph-1093862 Supplementary File S6.docx]

**Supplementary File S6.** Overview of the parcours of the progress activity including all FMS of the sessions

|  |  | **Proces evaluation question** | **Data sources** | **Tools** | **Timing/frequency** | **Data analysis** |
| --- | --- | --- | --- | --- | --- | --- |
| 1 | Fidelity (quality) | To what extent was the intervention implemented consistently with the underlying theory and philosphy? | Facilitators of the sessions | Process evaluation questionnaire (PEQ), questioning whether the facilitators think each intervention goal was included in the intervention | PEQ at the end of the program (for facilitators) | Describing/quantifying how much of the intended goals of the intervention were reached |
|  |  |  | Participants (fathers) | Process evaluation questionnaire (PEQ), questioning whether the fathers think each intervention goal was included in the intervention | PEQ at the end of the program (for fathers) | Describing/quantifying how much of the intended goals of the intervention were reached |
| 2 | Dose Delivered (completeness) | To what extent were all modules and units within the intervention/program implemented/delivered | Facilitators of the sessions | Self-reported checklist and observation with checklist of components included per session (information part, goal setting, active part with all subcomponents) | In each session, by all facilitators (3-4) | Describing/quantifying how much of the intended components of the intervention were given/delivered |
| 3a | Dose Received (exposure) | In what extent do the participants actively engage in the intervention and use the materials/components provided? | Participants (fathers) | Process evaluation questionnaire (PEQ) with checklist, questioning which of the components included per session (information part, goal setting, active part with all subcomponents) were used/performed | After each session, by all fathers | Describing/quantifying how much of the face-to-face part of the intervention was recieved |
|  |  |  | Main researcher (JL) | Analyzing log data, i.e. in which degree fathers used the website (profile) for logging goals and activities, and other aspects of the website | PEQ at the end of each session and at the end of the program (for fathers) | Describing/quantifying how much of the online part of the intervention was recieved |
|  |  |  | Main researcher (JL) | Initial use: Analyzing the log data, i.e. how often the participants enter their goals and activities on profile? | Log data during the entire intervention and in between two sessions | Describing/quantifying how much of the online part of the intervention was recieved |
|  |  |  | Main researcher (JL) | Continued use: Analyzing the log data, i.e. how often the participants enter their goals and activities on profile? | Log data after the program (when instructed to keep using it) | Describing/quantifying how much of the online part of the intervention was recieved, after the program |
| 3b | Dose Received (satisfaction) | Did fathers and children enjoy the intervention? Were they satisfied? | Facilitators of the sessions | Process evaluation questionnaire (PEQ), questioning in which degree fathers enjoyed and are satisfied about the intervention | PEQ at the end of each session and at the end of the program (for facilitators) | Bar chart of degree of fathers' and children's satisfaction (according to facilitators) |
|  |  |  | Participants (fathers) | Process evaluation questionnaire (PEQ), questioning in which degree fathers enjoyed and are satisfied about the intervention | PEQ at the end of each session and at the end of the program (for fathers) | Bar chart of degree of fathers' satisfaction (according to them) |
|  |  |  | Participants (children) | Process evaluation questionnaire (PEQ), questioning in which degree children enjoyed and are satisfied about the intervention - questioned with a PEQ tailored to their level, with emoji's | PEQ at the end of each session(for children) | Bar chart of degree of children's satisfaction (according to them) |
| 4 | Reach (participation rate) | What was the participation rate across all the session, and on average? | Facilitators of the sessions | Attendance, including documentation of barriers to participation | Each session | Look at attendance rate numbers |
| 5 | Recruitment | What procedures were followed to recruit fathers and children for the intervention? | Main researcher (JL) | Main researcher documents all recruitment activities | During recruitment phase | Decription of recruitment activities/procedures |
| 6 | Context | What were the barriers and facilitators for implenting the intervention? | Facilitators of the sessions | Process evaluation questionnaire (PEQ), questioning whether the facilitators think were the main barriers and facilitators during the intervention (both of the sessions and of the website/profile) | PEQ at the end of each session and at the end of the program (for facilitators) | Summative description of all barriers and facilitators for each of the components |
|  |  |  | Participants (fathers) | Process evaluation questionnaire (PEQ), questioning whether the fathers think were the main barriers and facilitators during the intervention (both of the sessions and of the website/profile) | PEQ at the end of each session and at the end of the program (for fathers) | Summative description of all barriers and facilitators for each of the components |
|  |  |  | Participants (children) | Process evaluation questionnaire (PEQ), questioning whether the children think were the main barriers and facilitators during the intervention (of the sessions) - questioned with a PEQ tailored to their level, with emoji's | PEQ at the end of each session (for children) | Summative description of all barriers and facilitators for each of the components |
