# Supplementary figures and images for: A Family-Based Lifestyle Intervention Focusing on Fathers and Their Children Using Co-Creation: Study Protocol of the Run Daddy Run Intervention
Source: Int J Environ Res Public Health. 2021 Feb 13;18(4):1830. doi: 10.3390/ijerph18041830 (PMC7918485; doi:10.3390/ijerph18041830)

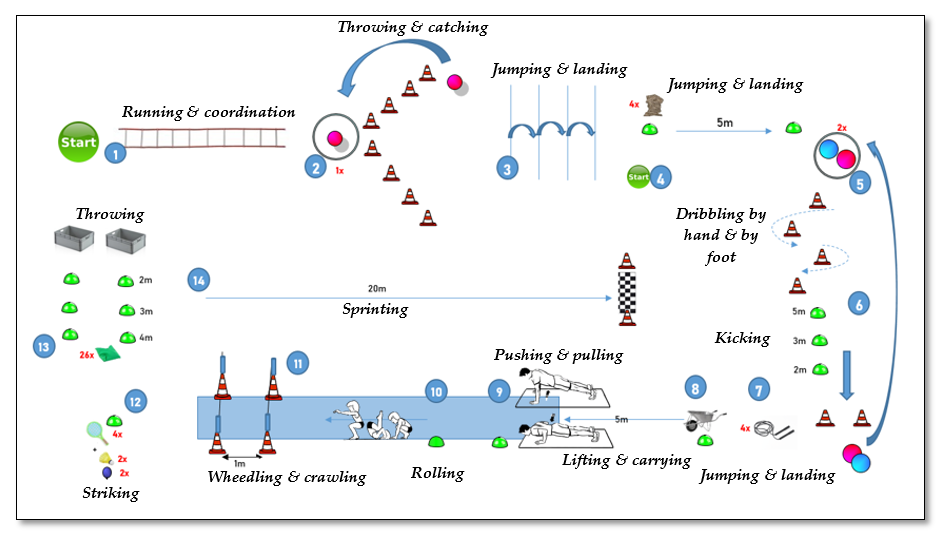

Supplement: Supplementary file 1 [file ijerph-18-01830-s001.zip › ijerph-1093862 Supplementary File S4.png]

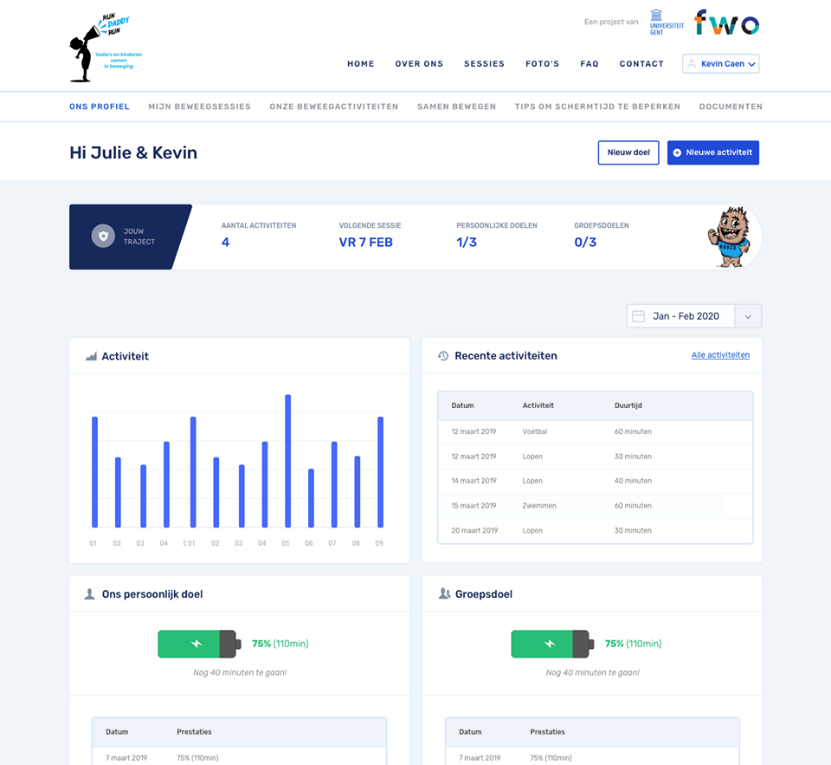

Supplement: Supplementary file 1 [file ijerph-18-01830-s001.zip › ijerph-1093862 Supplementary File S5.png]
